# *Citrus reticulata* Essential Oil as a Bioactive Food Ingredient: Composition Variability, Extraction, Functional Bioactivities, and Cross‐Study Quantitative Analysis

**DOI:** 10.1002/fsn3.72380

**Published:** 2026-09-21

**Authors:** Jiaojiao Zhang, Peng Zhou, Yongxian Chen, Andrea Brenciani, Jing Zhou, Iman Zarei, Amnah Nasim

**Affiliations:** ^1^ College of Food and Health Zhejiang A& F University Hangzhou China; ^2^ Biological Physics Group University of Manchester Manchester UK; ^3^ Institute of Process Engineering Chinese Academy of Sciences Beijing P. R. China; ^4^ Tea Resources Utilization and Quality Testing Key Laboratory of Sichuan Province Sichuan Agricultural University Chengdu China; ^5^ Department of Biomedical Sciences and Public Health Polytechnic University of Marche Ancona Italy; ^6^ Agriculture and Rural Affairs Bureau of Qujiang District Quzhou Zhejiang China; ^7^ Institute of Public Health and Clinical Nutrition, School of Medicine, Faculty of Health Science University of Eastern Finland Kuopio Finland; ^8^ Jeonbuk RICE Intelligence Innovation Research Center Jeonbuk National University Jeonju Jeonbuk Republic of Korea

**Keywords:** bioactive food ingredient, *Citrus reticulata*, composition variability, cross‐study quantitative analysis, essential oil, extraction technology

## Abstract

*Citrus reticulata* essential oil (CrEO) is a complex mixture of volatile and aroma‐active phytochemicals mainly obtained from the peel oil glands of *C. reticulata* . Its chemical composition is dominated by monoterpene hydrocarbons, particularly limonene, together with other representative constituents such as gamma‐terpinene, beta‐myrcene, alpha‐pinene, sabinene, terpinolene, oxygenated terpenoids, aldehydes, esters, alcohols, ketones, and minor aroma‐active compounds. As a citrus‐derived bioactive ingredient, CrEO has attracted increasing attention for its potential relevance to food preservation, functional product development, and the value‐added utilization of citrus processing resources. This review comprehensively summarizes the chemical composition and volatile profile of CrEO and discusses the major factors responsible for compositional variability, including plant tissue, fruit maturity, cultivar identity, geographic origin, extraction method, processing conditions, and storage. In addition, a standardized, literature‐derived secondary dataset is constructed to enable quantitative cross‐study comparison, visualization, and multivariate analysis of CrEO composition despite differences in sample sources, extraction methods, analytical approaches, and reporting units. The effects of hydrodistillation, cold pressing, and emerging extraction approaches on essential oil yield, compositional stability, and functional quality are also discussed. Reported biological activities of CrEO, especially antioxidant, antimicrobial, antifungal, anti‐inflammatory, and other food‐relevant bioactivities, are critically reviewed in relation to their potential roles in food preservation, natural flavoring, functional food formulation, and bioactive ingredient development. Current evidence gaps are further identified, including the need for standardized compositional reporting, stronger activity validation in real food systems, improved safety and sensory assessment, and application‐oriented studies. Overall, this review highlights the importance of integrating compositional characterization with standardized comparative analysis to support quality control, functional evaluation, and application‐oriented development of CrEO.

## Graphical abstract

Integrated framework of *Citrus reticulata* essential oil (CrEO), covering source tissues, key volatile constituents, extraction and processing factors, functional bioactivities, and standardized dataset‐based analysis of compositional variability.

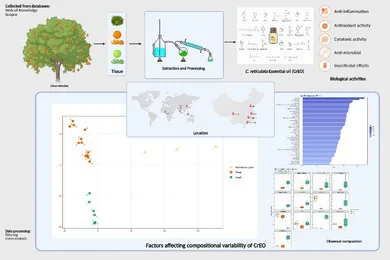

## 1 Introduction

*Citrus reticulata* , commonly known as mandarin orange, is an economically important species of the Rutaceae family and is widely cultivated worldwide for its distinctive flavor, aroma, and nutritional value (Olatunde et al. 2024; L. Li et al. 2024). In addition to its edible pulp, *C. reticulata* generates substantial peel residues during processing, and citrus peels may account for approximately 40%–50% of the total fruit mass (Singh et al. 2020). Although citrus peels are often treated as agro‐industrial by‐products, they are rich in diverse secondary metabolites, including volatile terpenoids and oxygenated compounds found in essential oils, as well as non‐volatile phenolic compounds (Singh et al. 2020). These constituents contribute to the antioxidant, antimicrobial, and other bioactive properties of citrus peels (Sarkar et al. 2026; T.‐X. Li et al. 2026). Therefore, the recovery and utilization of bioactive compounds from *C. reticulata* peel represent a promising strategy for improving the value‐added and sustainable use of citrus‐processing residues. Among these peel‐derived bioactive products, *C. reticulata* essential oil (CrEO) has attracted increasing attention because of its characteristic citrus aroma, complex volatile composition and diverse functional properties.

CrEO is typically dominated by limonene, a major monoterpene hydrocarbon that contributes substantially to its aromatic profile and biological properties (Kamal et al. 2011; Hu et al. 2024). In addition to limonene, CrEO contains a range of volatile constituents, including alpha‐pinene, beta‐myrcene, gamma‐terpinene, sabinene, linalool, citral isomers, and other terpenoids and oxygenated compounds (Krishnakumar et al. 2025). These constituents are closely associated with the sensory attribution, antioxidant (Hao et al. 2025), antimicrobial (Yabalak et al. 2022), antifungal (Namazi et al. 2025), anti‐inflammatory (Wen et al. 2024), insecticidal, and potential anticancer effects (Castro et al. 2026). From a food bioscience perspective, such properties support the potential use of CrEO as a natural flavoring material, food‐preservation agent, and bioactive ingredient for functional product development.

The chemical composition and functional properties of CrEO are strongly influenced by multiple intrinsic and extrinsic factors, including cultivar (Merle et al. 2004), genetic variation (Luro et al. 2023), fruit maturity, plant tissue (W. Zhang et al. 2025), geographical origin, and extraction methods. Peel‐derived CrEO is generally enriched in monoterpenes and other aroma‐active compounds, whereas oils obtained from leaves and flowers may display different profiles, often containing higher proportions of green‐note aldehydes or oxygenated monoterpenes (W. Zhang et al. 2025; Kasali et al. 2010). This compositional variability is important because it directly affects aroma quality, biological activity, stability, safety, and application performance. Therefore, a clear understanding of the relationship among botanical source, extraction conditions, chemical composition, and functional behavior is essential for the quality control and food‐oriented application of CrEO (Wolffenbüttel et al. 2015; Ishfaq et al. 2021; Xu et al. 2026).

The extraction technology is a critical factor influencing the yield, composition quality, and sustainability of CrEO production. Hydro‐distillation remains one of the most used techniques for extracting essential oils from citrus species, including *C. reticulata* (Mehmood et al. 2013). However, this conventional method is often associated with limitations such as prolonged extraction time and relatively low yield (R. Sharma 2024; Cui et al. 2021). To overcome these limitations, several advanced extraction strategies have been explored, including microwave‐ (Dao et al. 2020), ultrasound‐assisted (G. Li, Liu, et al. 2022; Song et al. 2022), deep eutectic solvent (DES)‐based methods (Chen et al. 2025). The integration of microwave or ultrasound technologies with green solvents such as deep eutectic solvents may further enhance mass transfer, reduce processing time, and improve extraction efficiency, thereby contributing to more sustainable essential oil recovery (R. Sharma 2024).

In recent years, the biological activities of CrEO have been extensively investigated. Its antioxidant capacity is frequently evaluated through assays such as DPPH, ABTS, and FRAP, which indicate its potential to scavenge free radicals and reduce oxidative deterioration (Mehmood et al. 2013; Xie et al. 2025). CrEO also exhibits antimicrobial activity against various pathogenic and spoilage‐related microorganisms, including *Escherichia coli* , *Staphylococcus aureus* , *Pseudomonas aeruginosa* , and *Candida albicans* , with activity often increasing in a concentration‐dependent manner (Khalaf et al. 2022) (Namazi et al. 2025; Yabalak et al. 2022). In addition, anti‐inflammatory effects, cytotoxic activity against breast cancer cell lines such as MCF‐7 and MDA‐MB‐231 (Job et al. 2024), and insecticidal effects against mosquito species such as *Culex pipiens* and *Aedes albopictus* have been reported (Shaw et al. 2023; J. Cao et al. 2024). Although these findings suggest that CrEO has considerable potential as a natural bioactive agent, its practical application in food systems still requires further validation, particularly with respect to activity retention, sensory acceptability, safety, stability, and interactions with food matrices.

Despite this growing body of research on CrEO, current knowledge remains fragmented. Existing studies often focus on individual aspects, such as extraction yield, chemical composition, antimicrobial activity, antioxidant capacity, or specific applications, whereas relatively few studies have systematically integrated these findings from a compound‐level perspective. In particular, the relationships among extraction method, compositional variation, bioactivity, and practical application require further clarification. Moreover, although delivery systems such as microemulsions and nanoencapsulation have been developed to improve the stability, bioavailability, controlled release, and safety of CrEO, their relevance to different food and functional product applications has not been fully synthesized (Prommaban and Chaiyana 2022; Torshabi et al. 2023).

Therefore, this review aims to provide a systematic and food‐oriented synthesis of current knowledge concerning CrEO, with particular emphasis on its chemical composition, extraction and processing methods, biological activities, and potential applications. It summarizes the major biological activities reported for CrEO, particularly its antioxidant, antifungal effects, and considers their relevance to food preservation, natural flavoring, and functional product development. By integrating evidence from existing studies, the review also characterizes the major constituents of CrEO and critically examines compositional variability associated with geographical origin, tissue type, cultivar or genotype, fruit maturity, extraction method, analytical approach and reporting unit.

Beyond a conventional narrative synthesis, this review constructs a standardized, literature‐derived secondary dataset to enable quantitative cross‐study comparisons, visualization, and multivariate analysis of CrEO composition. Tissue‐level comparisons, heatmap visualization, and partial least squares discriminant analysis (PLS‐DA) are used to characterize compositional patterns and identify the compounds contributing most strongly to tissue differentiation. This data‐driven approach provides a comparative framework for understanding the compositional variability and potential chemical markers of CrEO. Finally, the review identifies current evidence gaps and future research priorities, including standardized compositional reporting, optimized extraction strategies, investigation of synergistic interactions among peel‐derived volatile constituents, validation of biological activity in real food systems, comprehensive safety and sensory evaluation, and the scalable valorization of CrEO and *C. reticulata* peel resources for sustainable, high‐value food and related applications.

## 2 Chemical Composition of Peel‐ and Leaf‐Derived CrEO

### 2.1 Literature Search and Construction of the Secondary Dataset

Relevant publications were searched in the Web of Science Core Collection and Scopus on [SEARCH DATE]. The Web of Science search query was: TS = (“ *Citrus reticulata* ”) AND TS = (pericarp OR peel OR rind OR skin) AND TS = (processing OR extraction OR manufacturing OR preparation) AND TS = (“volatile compounds” OR “essential oils” OR “aromatic compounds” OR fragrance) AND TS = (extraction OR analysis OR composition OR characterization). For Scopus, the query was adapted to the title, abstract, and keyword fields as follows: TITLE‐ABS‐KEY(“ *Citrus reticulata* ”) AND TITLE‐ABS‐KEY(pericarp OR peel OR rind OR skin) AND TITLE‐ABS‐KEY(processing OR extraction OR manufacturing OR preparation) AND TITLE‐ABS‐KEY(“volatile compounds” OR “essential oils” OR “aromatic compounds” OR fragrance) AND TITLE‐ABS‐KEY(extraction OR analysis OR composition OR characterization). A complementary search was conducted in the China National Knowledge Infrastructure (CNKI) using corresponding Chinese keywords to identify relevant Chinese‐language studies. Additional publications were identified by examining the reference lists of eligible studies.

The titles, abstracts, and full texts of the retrieved publications were screened. Studies were included when they reported clear compound‐level compositional data for essential oils or volatile fractions derived from *C. reticulata* peel or leaves. Studies were excluded when they did not provide usable compositional data, when the plant species or tissue could not be clearly identified, or when they duplicated data reported in another publication. The final dataset comprised 10 eligible studies and 24 compositional profiles.

To support a quantitative comparison of CrEO composition across published studies, a structured secondary dataset was compiled from the reviewed literature. Compound‐level data were extracted from original tables, including reference, tissue type, maturity stage, geographic origin, extraction or sampling method, analytical method, reported value, and reported unit. Because several publications reported multiple oils, each literature‐derived sample was retained as an independent observation, and sample identifiers were constructed from the reference and sample descriptors.

Compound names were standardized conservatively to improve cross‐study comparability. Orthographic variants and clear synonyms were merged where appropriate, for example, D‐limonene was standardized as limonene, 1‐beta‐pinene as beta‐pinene, and alpha/beta‐myrcene variants as myrcene when the reported information supported such grouping. In contrast, clearly distinct geometric or structural isomers were retained as separate compounds. Reported values were then converted into a normalized relative composition variable for quantitative comparison. Percentage‐based values were treated as relative composition data, whereas mg/g values were converted to within‐sample relative percentages. Normalized composition data were used for visualization and multivariate analysis.

### 2.2 PLS‐DA Input Matrix, Model Construction, and Validation

The standardized compound‐level dataset was transformed from long to wide format for partial least squares discriminant analysis (PLS‐DA). Each compositional profile constituted one row, each standardized compound constituted one predictor variable, and tissue type constituted the categorical response variable. The initial input matrix contained 24 compositional profiles and 168 compound variables. Variables with zero variance across all profiles were excluded because they could not contribute to discrimination. After removing 12 zero‐variance variables, the final PLS‐DA input matrix contained 24 observations and 156 compound variables.

PLS‐DA was performed in R version 4.2.0 using the ropls package (version 1.24.0). Predictor variables were mean‐centered and scaled to unit variance using standard scaling (scaleC = “standard”). Models containing one, two, and three predictive components were initially compared using seven‐fold cross‐validation and 100 permutation tests. No orthogonal components were included (orthoI = 0), ensuring that the analysis represented PLS‐DA rather than OPLS‐DA. The random seed was set to 123 to support reproducibility.

Based on the balance between predictive performance, interpretability, and model parsimony, the two‐component model was selected as the primary model (predI = 2). The selected model was subsequently subjected to a more extensive final validation using 200 permutation tests. The final model produced cumulative R^2^X, R^2^Y, and Q^2^ values of 0.302, 0.871, and 0.772, respectively, with a root mean square error of estimation (RMSEE) of 0.170. The permutation‐test *p*‐values were 0.005 for both R^2^Y and *Q* ^2^, indicating that the observed discrimination was unlikely to result from random class assignment.

Variable importance in projection (VIP) scores were extracted from the final two‐component model. Compounds with VIP > 1 were considered important contributors to tissue‐level compositional discrimination. The PLS‐DA score plot was used to visualize the relative separation among peel, leaf, and immature peel profiles, whereas the VIP analysis was used to identify compounds contributing most strongly to this separation. Given the limited number of compositional profiles, unequal group sizes, heterogeneous analytical methods, and literature‐derived nature of the dataset, the PLS‐DA results were interpreted as exploratory evidence of compositional differentiation rather than as a confirmatory classification model.

### 2.3 Limonene as the Dominant Compound of Peel‐Derived CrEO

**FIGURE 1:**
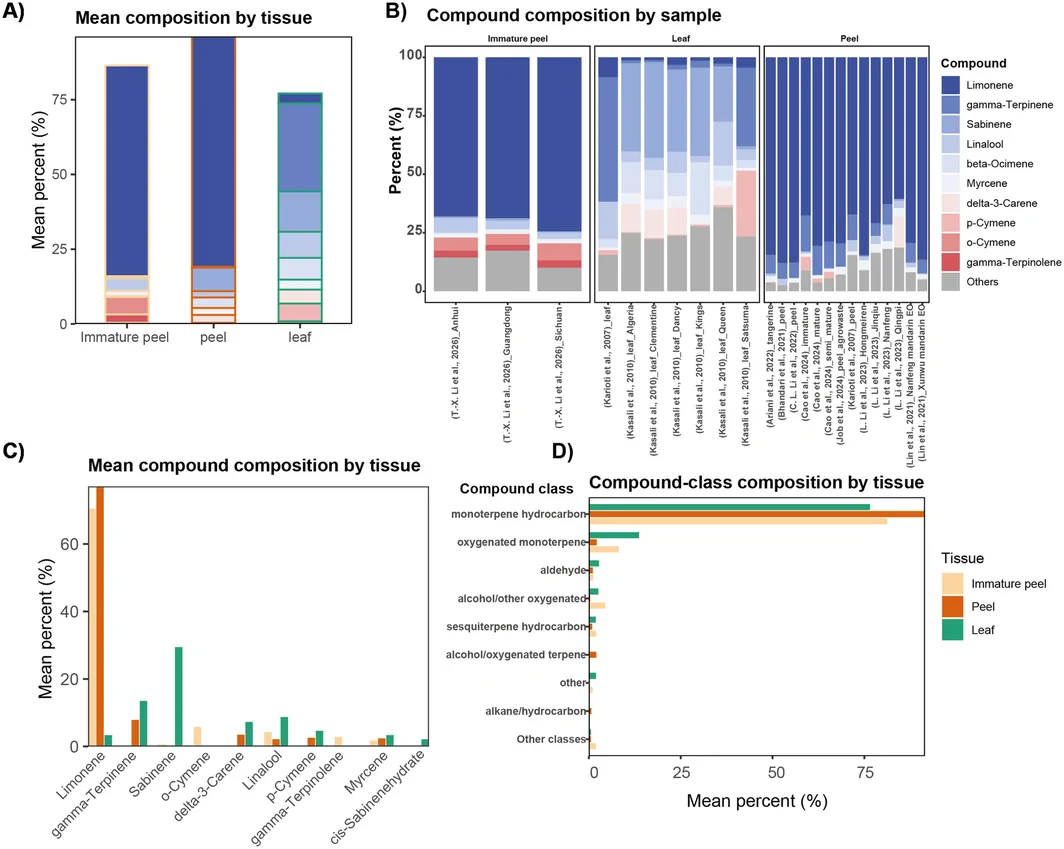
Tissue‐ and sample‐level composition of *Citrus reticulata* essential oils. (A) Mean composition by tissue Stacked bar plot showing mean compound composition by tissue group based on normalized relative abundance. The top compounds are shown individually, and all remaining compounds are grouped as “Others”. Peel and immature peel samples were dominated by Limonene, whereas leaf samples showed a more chemically diverse profile enriched in sabinene, γ‐terpinene, β‐ocimene, linalool, δ‐3‐carene, and p‐cymene. (B) Sample‐level composition Sample‐level stacked bar plot showing compound composition across individual literature‐derived samples. Samples are grouped by tissue type. Each sample_id combines the literature source and tissue/sample identity. Peel‐derived samples were generally Limonene‐rich, whereas leaf samples showed greater inter‐sample variation and included sabinene‐rich, γ‐terpinene‐rich, and p‐cymene‐rich chemotypes. (C) Mean major compounds by tissue Grouped bar plot showing mean normalized abundance of selected high‐contribution compounds across tissue groups. Tissue colors represent immature peel, peel, and leaf. The plot highlights Limonene enrichment in peel‐related samples and enrichment of sabinene, γ‐terpinene, β‐ocimene, linalool, δ‐3‐carene, and p‐cymene in leaf samples. (D) Compound‐class composition by tissue Compound‐class composition by tissue group. Compound percentages were first summed by compound class within each sample and then averaged within each tissue group. Major compound classes are shown individually, whereas low‐abundance classes were grouped as “Other classes.” Monoterpene hydrocarbons dominated all tissue groups, especially peel samples, while leaf samples showed higher contributions from oxygenated monoterpenes and minor compound classes. Immature peel samples.

Limonene was the dominant constituent of peel‐derived and immature peel‐derived *C. reticulata* essential oils (CrEOs). Across the normalized dataset, Limonene showed the highest overall mean relative abundance, accounting for 54.48% across all 24 literature‐derived samples. Its distribution was strongly tissue dependent. At the tissue level, Limonene was highly abundant in peel and immature peel samples, with mean normalized relative abundances of 76.65% and 70.44%, respectively, whereas leaf samples contained only 3.32% on average (Figure 1A).

The sample‐level composition plot confirmed that Limonene was the principal compound in most peel‐derived oils (Figure 1B). Among percentage‐based peel oils, L. Li, Jiang, et al. (2023) reported the lowest originally reported Limonene value in Qingpi peel oil (58.38%), whereas C. L. Li, Cai, et al. (2022) reported the highest value in a Chinese *C. reticulata* peel oil sample (86.66%). After within‐sample normalization, peel‐derived Limonene showed a similar range, from 58.38% in Qingpi peel oil (L. Li, Jiang, et al. 2023) to 87.91% in Nepalese mandarin peel oil (Bhandari et al. 2021).

Other peel‐derived oils were also Limonene‐rich. C. L. Li, Cai, et al. (2022) reported 87.80% normalized Limonene in a Chinese *C. reticulata* peel oil sample, Lin et al. (2021) reported 86.54% in Xunwu mandarin EO and 79.42% in Nanfeng mandarin EO, Ariani et al. (2022) reported 84.52% in Indonesian tangerine peel oil, L. Li, Jiang, et al. (2023) reported 83.49% in Hongmeiren, 69.92% in Jinqiu, and 61.18% in Nanfeng peel oils, Cao et al. (2024) reported 80.60% in mature Chachi peel EO, Job et al. (2024) reported 79.65% in Indian agrowaste‐derived peel oil, and Karioti et al. (2007) reported 67.27% in Nigerian unripe peel oil.

Fruit maturity also affected the peel volatile profile. In the Chachi peel series, Cao et al. (2024) reported that normalized Limonene increased from 67.57% in immature peel to 78.80% in semi‐mature peel and 80.60% in mature peel. The immature peel samples from Anhui, Guangdong, and Sichuan reported by T.‐X. Li et al. (2026) were originally expressed in mg/g rather than percentage, with Limonene concentrations of 96.468, 89.698, and 65.246 mg/g, respectively. After normalization, these samples remained Limonene‐dominant, with relative abundances of 68.06%, 68.84%, and 74.42%, respectively.

By contrast, leaf oils were not Limonene‐dominant. Karioti et al. (2007) reported a Nigerian leaf oil with only 8.53% normalized Limonene, while Kasali et al. (2010) reported six Nigerian mandarin leaf oils with much lower normalized Limonene levels, ranging from 1.34% to 4.49%. These values contrast sharply with the Limonene‐rich peel and immature peel samples, supporting the interpretation of Limonene as a peel‐related marker rather than a universal marker of all CrEO samples.

### 2.4 Other Characteristic Constituents

Besides Limonene, several recurrent monoterpenes and oxygenated terpenoids were identified across the analyzed samples. Across all samples, the major non‐Limonene constituents were γ‐terpinene with an overall mean of 8.50%, sabinene at 7.52%, linalool at 4.22%, β‐ocimene at 3.01%, myrcene at 2.49%, δ‐3‐carene at 2.37%, p‐cymene at 1.79%, terpinen‐4‐ol at 1.67%, β‐pinene at 1.56%, and α‐pinene at 1.18%. These compounds formed the main secondary fraction of CrEO composition (Figure 1A–C). Together, these compounds represented the main secondary fraction of CrEO composition.

γ‐Terpinene was an important secondary monoterpene in peel oils and in some leaf oils. At the tissue level, γ‐terpinene averaged 7.83% in peel samples and 13.49% in leaf samples, whereas it was absent from the immature peel samples in the standardized compound matrix. In peel‐derived oils, Karioti et al. (2007) reported 10.84% normalized γ‐terpinene in Nigerian peel oil, Bhandari et al. (2021) reported 6.76% in Nepalese mandarin peel oil, and C. L. Li, Cai, et al. (2022) reported 6.46% in a Chinese *C. reticulata* peel oil sample. Lin et al. (2021) reported 8.22% and 5.83% γ‐terpinene in Nanfeng and Xunwu mandarin EOs, respectively, while Ariani et al. (2022) reported 7.80% in Indonesian tangerine peel oil and Job et al. (2024) reported 9.86% in agrowaste‐derived peel oil. In the Chachi maturity series, Cao et al. (2024) reported 15.45% γ‐terpinene in immature peel, 11.34% in semi‐mature peel, and 12.42% in mature peel. Leaf oils showed stronger variability: Karioti et al. (2007) reported 53.16% γ‐terpinene in a Nigerian leaf oil, whereas Kasali et al. (2010) reported 33.44% in the Satsuma leaf oil and much lower levels in other Nigerian mandarin leaf cultivars. These results indicate that some leaf oils are γ‐terpinene‐rich rather than sabinene‐rich.

Sabinene showed the strongest leaf‐associated pattern among the major monoterpenes. Its mean normalized abundance was 25.23% in leaf samples, compared with only 0.65% in immature peel samples and 0.14% in peel samples. In the leaf‐oil dataset reported by Kasali et al. (2010), five Nigerian mandarin leaf cultivars were sabinene‐rich, with normalized values ranging from 23.59% to 40.93%. By contrast, peel oils contained only trace to low sabinene levels. C. L. Li, Cai, et al. (2022) reported 0.03% sabinene in a Chinese *C. reticulata* peel oil sample, Lin et al. (2021) reported 0.08% and 0.35% in Xunwu and Nanfeng mandarin EOs, respectively, and Ariani et al. (2022) reported 0.45% in Indonesian tangerine peel oil. In the peel cultivar dataset reported by L. Li, Jiang, et al. (2023), sabinene ranged from 0.34% to 0.69%. The immature peel samples from Anhui, Guangdong, and Sichuan reported by T.‐X. Li et al. (2026) also contained low normalized sabinene values, ranging from 0.59% to 0.91%. This strong tissue contrast supports sabinene as a leaf‐enriched marker (Figure 1A–C; Table 1).

**TABLE 1:** Integrated biomarker table.

Compound	Main enriched tissue	Mean in immature peel (%)	Mean in peel (%)	Mean in leaf (%)	VIP	FDR‐*p*	KW *ε* ^2^	Interpretation
Limonene	Peel/Immature peel	70.44	76.65	3.32	2.26	0.0124	0.625	Strong peel‐related marker
Terpinolene	Leaf	0.29	0.28	2.28	2.07	0.0124	0.62	Strong leaf‐associated marker
α‐Terpinene	Leaf	0	0.17	0.9	1.83	0.0145	0.506	Leaf‐associated marker; detected in two tissue groups
Sabinene	Leaf	0.65	0.14	25.23	1.81	0.0124	0.772	Strongest leaf‐enriched discriminant
β‐Caryophyllene	Leaf	0	0.04	1.06	1.66	0.0129	0.675	Leaf‐associated sesquiterpene
β‐Pinene	Leaf	0.66	0.85	3.35	1.63	0.0124	0.585	Leaf‐enriched monoterpene
Carvone	Immature peel	0.44	0.03	0	1.6	0.0447	0.595	Immature peel‐associated marker
α‐Thujene	Leaf	0	0.14	0.83	1.6	0.0129	0.622	Leaf‐associated marker; detected in two tissue groups
α‐Pinene	Leaf	0	0.82	2.4	1.55	0.0396	0.372	Leaf‐enriched but also present in peel
Linalool	Leaf/Immature peel	4.29	1.96	8.72	1.53	0.0474	0.29	Significant but less tissue‐specific
Terpinen‐4‐ol	Leaf	1.98	0.72	3.43	1.4	0.0474	0.304	Leaf‐enriched with within‐group variation
Citronellal	Leaf	0	0.05	0.95	1.22	0.0243	0.641	Leaf‐associated oxygenated monoterpenoid
β‐Ocimene	Leaf	0.22	0.03	10.17	1.08	0.0396	0.739	Strong leaf‐enriched compound; influenced by high‐value leaf samples

*Note:* VIP values were obtained from the PLS‐DA model (Figure S2). FDR‐*p* values were derived from Kruskal‐Wallis tests followed by FDR correction. KW *ε* ^2^ represents Kruskal‐Wallis epsilon‐squared effect size.

Linalool was present in all three tissue categories but was most abundant in leaf samples, with mean normalized relative abundances of 8.72% in leaf, 4.29% in immature peel, and 1.96% in peel samples. The highest leaf values occurred in the Nigerian leaf oil reported by Karioti et al. (2007) and the Queen leaf oil reported by Kasali et al. (2010). Peel samples generally had lower linalool levels, although L. Li, Jiang, et al. (2023) reported a relatively high value of 6.78% in Nanfeng peel oil. In the immature peel samples from Anhui, Guangdong, and Sichuan, T.‐X. Li et al. (2026) reported linalool concentrations of 2.402–8.996 mg/g, corresponding to normalized relative abundances of 2.74%–6.35%. Thus, linalool contributed to tissue discrimination, but it was less tissue‐specific than limonene or sabinene.

β‐Ocimene, δ‐3‐carene, and p‐cymene further contributed to the heterogeneity of leaf oils. β‐Ocimene averaged 10.17% in leaf samples but was nearly absent from peel samples. Its high leaf contribution was mainly associated with the Nigerian mandarin leaf oils reported by Kasali et al. (2010) especially the Kings leaf oil, together with lower values in the Karioti leaf oil (Kasali et al. 2010; Karioti et al. 2007). δ‐3‐Carene averaged 6.22% in leaf samples and was high in several Kasali leaf cultivars, although Qingpi peel also contained a high δ‐3‐carene value of 12.66%, indicating cultivar‐specific accumulation (Kasali et al. 2010; L. Li, Jiang, et al. 2023). p‐Cymene averaged 4.64% in leaf samples, mainly because the Kasali Satsuma leaf sample contained 27.93% p‐cymene (Kasali et al. 2010). These results show that leaf‐derived CrEOs are chemically more heterogeneous than peel‐derived CrEOs.

Myrcene was a recurrent moderate‐abundance monoterpene, with tissue means of 1.77% in immature peel, 2.20% in peel, and 3.37% in leaf. In peel‐derived oils, Karioti et al. (2007) reported 1.41% normalized myrcene in Nigerian unripe peel oil, C. L. Li, Cai, et al. (2022) reported 1.80% in a Chinese *C. reticulata* peel oil sample, and Ariani et al. (2022) reported 2.43% in Indonesian tangerine peel oil. Job et al. (2024) reported a higher value of 3.32% in Indian agrowaste‐derived peel oil, while Lin et al. (2021) reported 2.09% and 2.03% in Nanfeng and Xunwu mandarin EOs, respectively. In the peel cultivar dataset of L. Li, Jiang, et al. (2023), myrcene ranged from 2.93% to 4.02%. Leaf values were mainly contributed by the Karioti and Kasali leaf‐oil datasets, with Karioti et al. (2007) reporting 1.20% in Nigerian leaf oil and Kasali et al. (2010) reporting 1.33%–4.80% across six Nigerian mandarin leaf oils. In the immature peel samples, myrcene accounted for 1.68%–1.85% of the recalculated relative composition based on the data reported by T.‐X. Li et al. (2026).

β‐Pinene and α‐pinene were generally minor in peel oils but were higher in leaf samples. β‐Pinene averaged 3.35% in leaf, compared with 0.85% in peel and 0.66% in immature peel. In leaf oils, Karioti et al. (2007) reported 4.31% β‐pinene in Nigerian leaf oil, while Kasali et al. (2010) reported 1.46%–7.65% across Nigerian mandarin leaf cultivars. In peel oils, β‐pinene was generally lower: C. L. Li, Cai, et al. (2022) reported 0.48%, Ariani et al. (2022) reported 0.72%, Karioti et al. (2007) reported 0.80%, and Cao et al. (2024) reported 0.94%–1.00% across Chachi peel maturity stages. Lin et al. (2021) reported 2.01% in Nanfeng mandarin EO and 0.46% in Xunwu mandarin EO, while L. Li, Jiang, et al. (2023) reported 0.05%–1.76% across four peel cultivars. In immature peel samples, β‐pinene accounted for 0.59%–0.80% of the recalculated relative composition based on the data reported by T.‐X. Li et al. (2026).

α‐Pinene showed a similar but slightly weaker leaf‐associated pattern. It averaged 2.40% in leaf and 0.82% in peel but was not detected in the immature peel samples in the standardized matrix. Karioti et al. (2007) reported 2.81% normalized α‐pinene in Nigerian leaf oil, and Kasali et al. (2010) reported 1.15%–3.98% across Nigerian mandarin leaf cultivars. In peel‐derived oils, α‐pinene was reported at 0.60% in Nigerian unripe peel oil (Karioti et al. 2007), 1.50% in the Chinese *C. reticulata* peel oil sample (C. L. Li, Cai, et al. 2022), 1.06% in Indonesian tangerine peel oil (Ariani et al. 2022), and 3.19% in Indian agrowaste‐derived peel oil (Job et al. 2024). Lin et al. (2021) reported 1.22% in Nanfeng mandarin EO and 0.83% in Xunwu mandarin EO, while Cao et al. (2024) reported 0.94%–1.08% across Chachi peel maturity stages.

### 2.5 Minor and Trace Aroma‐Active Constituents

Minor and trace constituents also contributed to the chemical diversity of CrEO. This fraction included aliphatic aldehydes, terpenoid aldehydes, oxygenated monoterpenes, phenolic compounds, and other aroma‐active constituents. Although these compounds were generally less abundant than limonene, γ‐terpinene, and sabinene, several showed tissue‐associated patterns.

Octanal and decanal were mainly associated with peel and immature peel samples and generally occurred as low‐abundance aldehydes. In immature peel samples, T.‐X. Li et al. (2026) originally reported octanal in mg/g, with concentrations of 0.443, 0.344, and 0.252 mg/g in Anhui, Guangdong, and Sichuan, respectively; after normalization, these corresponded to 0.31%, 0.26%, and 0.29%. Among peel oils, Karioti et al. (2007) reported the highest normalized octanal value, 1.61%, in Nigerian unripe peel oil. L. Li, Jiang, et al. (2023) reported octanal values of 0.58%–0.87% in the peel cultivar dataset where detected, while Ariani et al. (2022) reported 0.51% in Indonesian tangerine peel oil, Cao et al. (2024) reported 0.29%–0.69% across Chachi peel maturity stages, and Lin et al. (2021) reported 0.29% in Nanfeng mandarin EO.

Decanal showed a similar low‐abundance peel/immature peel‐associated pattern. In immature peel samples, T.‐X. Li et al. (2026) originally reported decanal concentrations of 0.847, 0.302, and 0.172 mg/g in Anhui, Guangdong, and Sichuan, respectively; after normalization, these corresponded to 0.60%, 0.23%, and 0.20%. In peel‐derived oils, L. Li, Jiang, et al. (2023) reported decanal values ranging from 0.02% in Qingpi to 0.83% in Jinqiu. Bhandari et al. (2021) and Karioti et al. (2007) both reported 0.40% decanal in Nepalese mandarin peel oil and Nigerian unripe peel oil, respectively, while Cao et al. (2024) reported 0.18% in Chachi immature peel oil, Job et al. (2024) reported 0.50% in Indian agrowaste‐derived peel oil, and Lin et al. (2021) reported 0.57% in Nanfeng mandarin EO. These aldehydes therefore occurred mainly as low‐abundance peel‐related volatiles.

Citronellal showed a clearer leaf‐associated pattern. It was low in peel samples: Bhandari et al. (2021) reported 0.11% normalized citronellal in Nepalese mandarin peel oil, Karioti et al. (2007) reported 0.30% in Nigerian unripe peel oil, Cao et al. (2024) reported 0.09% in Chachi immature peel oil, and Lin et al. (2021) reported only 0.01% in Xunwu mandarin EO. In the peel cultivar dataset of L. Li, Jiang, et al. (2023), citronellal was absent from Qingpi and ranged from 0.03% to 0.08% in the other peel cultivars. By contrast, Kasali et al. (2010) reported higher citronellal values in several Nigerian mandarin leaf oils, particularly Kings and Queen leaf oils, with normalized values of 2.50% and 3.13%, respectively. This leaf‐associated pattern was supported by the integrated biomarker analysis, where citronellal had VIP = 1.22, FDR‐adjusted *p* = 0.0243, and KW *ε* ^2^ = 0.641, indicating a statistically significant and large tissue‐associated effect (Table 1).

Citral‐related aldehydes were more prominent in selected leaf oils than in most peel oils. In the leaf‐oil dataset of Kasali et al. (2010), geranial reached 5.64% and neral reached 4.28% in the Queen leaf oil, while lower values were observed in other leaf cultivars. By contrast, peel oils generally contained only trace levels of these aldehydes: Karioti et al. (2007) reported 0.10% geranial and 0.10% neral in Nigerian unripe peel oil, Bhandari et al. (2021) reported 0.01% neral in Nepalese mandarin peel oil, and C. L. Li, Cai, et al. (2022) reported 0.21% neral in a Chinese *C. reticulata* peel oil sample.

Sinensal‐type aldehydes showed variable tissue associations. Kasali et al. (2010) reported α‐sinensal at 0.31%–2.06% and β‐sinensal at 0.79%–1.65% across Nigerian mandarin leaf oils, indicating a leaf‐associated tendency. In immature peel samples, T.‐X. Li et al. (2026) reported β‐sinensal at 0.552 and 0.439 mg/g in Anhui and Guangdong, respectively, corresponding to 0.39% and 0.34% after normalization. The same study also reported sinensal at 0.613, 0.484, and 0.077 mg/g in Anhui, Guangdong, and Sichuan immature peel samples, corresponding to 0.43%, 0.37%, and 0.09% after normalization. In peel oils, sinensal was detected at 0.01% in Jinqiu and 0.35% in Hongmeiren in the L. Li, Jiang, et al. (2023) cultivar dataset, while Karioti et al. (2007) reported 0.50% α‐sinensal in Nigerian unripe peel oil. Although β‐sinensal and related sinensal‐type aldehydes contributed to tissue variation, they did not meet the strict FDR‐adjusted *p* < 0.05 threshold in the final biomarker table and were therefore not classified among the strongest confirmed discriminant markers.

Phenolic and aromatic trace compounds were detected sporadically. Bhandari et al. (2021) reported methyl salicylate at 0.01% and thymol methyl ether at 0.11% in Nepalese mandarin peel oil. Thymol was detected in several peel samples: Bhandari et al. (2021) reported 0.08% in Nepalese mandarin peel oil, Lin et al. (2021) reported 0.18% in Nanfeng mandarin EO, and L. Li, Jiang, et al. (2023) reported 0.07%, 1.79%, and 0.76% in Qingpi, Nanfeng, and Jinqiu peel oils, respectively. Kasali et al. (2010) also reported 0.31% thymol in Queen leaf oil. Although these compounds occurred at low abundance, they may still contribute to aroma because many aldehydes, phenols, and oxygenated terpenoids have low odor thresholds.

### 2.6 Factors Affecting Compositional Variability of CrEO

The chemical composition of CrEO varied strongly with tissue type, as well as with cultivar, maturity stage, and geographic origin. Tissue type was the clearest source of variation. Peel‐derived oils were generally Limonene‐dominant, as shown in peel samples from Nigerian unripe peel oil (Karioti et al. 2007), Nepalese mandarin peel oil (Bhandari et al. 2021), Chinese *C. reticulata* peel oil (C. L. Li, Cai, et al. 2022), Nanfeng and Xunwu mandarin EOs (Lin et al. 2021), Indonesian tangerine peel oil (Ariani et al. 2022), the L. Li peel cultivar dataset (L. Li, Jiang, et al. 2023), Chachi peel oils across maturity stages (J. Cao et al. 2024), and Indian agrowaste‐derived peel oil (Job et al. 2024). Immature peel samples from Anhui, Guangdong, and Sichuan were also Limonene‐dominant after normalization Li et al. (2026). By contrast, leaf oils showed more diverse monoterpene and sesquiterpene profiles. Karioti et al. (2007) reported a γ‐terpinene/linalool‐rich Nigerian leaf oil, whereas Kasali et al. (2010) reported several sabinene‐rich Nigerian mandarin leaf oils and a Satsuma leaf oil enriched in γ‐terpinene and p‐cymene. Overall, leaf samples were enriched in sabinene, γ‐terpinene, β‐ocimene, linalool, δ‐3‐carene, p‐cymene, myrcene, β‐pinene, and α‐pinene, whereas peel and immature peel samples were characterized primarily by high Limonene abundance (Figure 1A–C).

At the compound‐class level, monoterpene hydrocarbons dominated all tissue groups, averaging 91.47% in peel, 81.22% in immature peel, and 76.53% in leaf samples. Leaf samples showed a higher oxygenated monoterpene fraction than peel samples (13.68% vs. 2.15%), whereas immature peel samples showed intermediate oxygenated monoterpene abundance (8.15%) and a relatively higher alcohol/other oxygenated fraction (4.44%) than peel samples. This class‐level pattern supports tissue‐dependent differences not only at the individual‐compound level but also at the broader chemical‐class level (Figure 1D).

**FIGURE 2:**
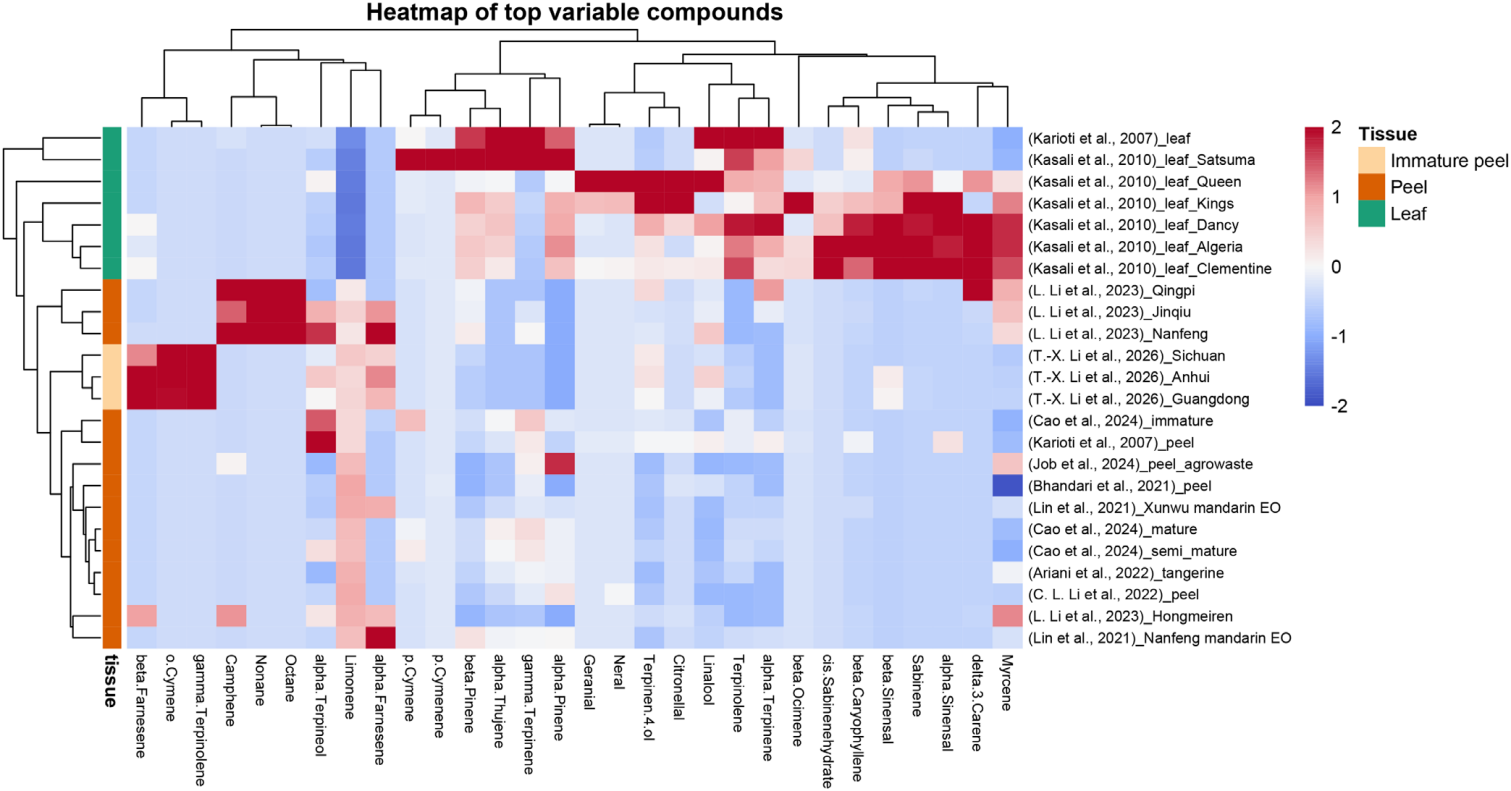
Heatmap of top variable compounds across CrEO samples. The heatmap shows standardized abundances of the most variable compounds across literature‐derived samples. Rows represent samples and columns represent compounds. Sample annotations indicate tissue type. Peel and immature peel samples were generally characterized by high limonene abundance, whereas leaf samples clustered separately and were enriched in sabinene, γ‐terpinene, β‐ocimene, linalool, δ‐3‐carene, p‐cymene, and related monoterpenes. The heatmap supports tissue‐dependent chemical differentiation and also reveals sample‐level heterogeneity within leaf and peel groups.

The heatmap of top variable compounds supported this tissue‐dependent structure. Peel and immature peel samples clustered with high limonene abundance, whereas leaf samples formed a chemically distinct group characterized by enrichment of sabinene, γ‐terpinene, β‐ocimene, linalool, δ‐3‐carene, p‐cymene, and related monoterpenes (Figure 2). The sample‐level bar plot further showed that leaf oils were more heterogeneous than peel oils (Figure 1B). Karioti et al. (2007) reported a Nigerian leaf oil characterized by high γ‐terpinene and linalool, whereas Kasali et al. (2010) reported a Satsuma leaf oil enriched in γ‐terpinene and p‐cymene. In the same Kasali leaf‐oil dataset, several other Nigerian mandarin leaf cultivars were sabinene‐rich. These patterns indicate that leaf‐derived CrEOs cannot be represented by a single chemotype.

**FIGURE 3:**
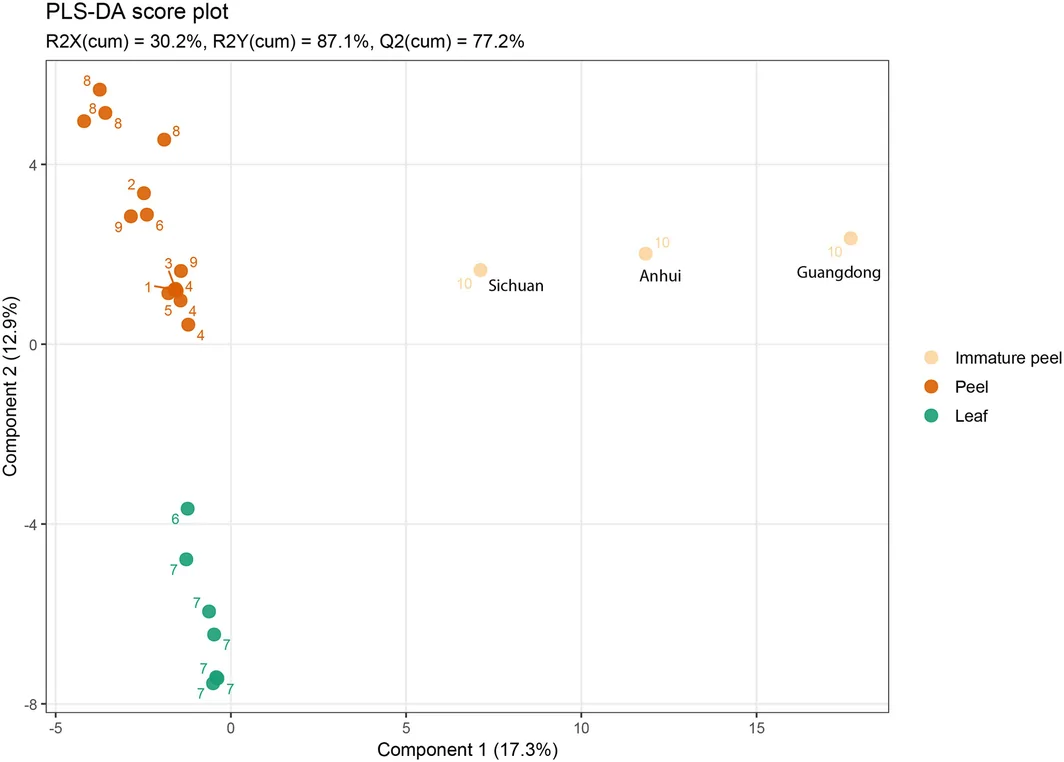
PLS‐DA score plot of CrEO samples based on normalized volatile composition. The PLS‐DA model was constructed using standardized predictor variables, with tissue group specified as the response variable. The final model comprised two predictive components and no orthogonal component. Component 1 and Component 2 explained 17.3% and 12.9% of the X variance, respectively, resulting in cumulative model statistics of R^2^X(cum) = 30.2%, R^2^Y(cum) = 87.1%, and *Q* ^2^(cum) = 77.2%. The score plot showed clear discrimination among tissue groups. Immature peel samples were separated primarily along Component 1, whereas leaf samples were distinguished from peel‐related samples mainly along Component 2. The numbered labels in the PLS‐DA score plot denote the source studies as follows: 1 = Ariani et al. (2022); 2 = Bhandari et al. (2021); 3 = C. L. Li, Cai, et al. (2022); 4 = Cao et al. (2024); 5 = Job et al. (2024); 6 = Karioti et al. (2007); 7 = Kasali et al. (2010); 8 = L. Li, Jiang, et al. (2023); 9 = Lin et al. (2021); and 10 = T.‐X. Li et al. (2026).

PLS‐DA further confirmed tissue‐dependent separation in volatile composition. The model used two predictive components and no orthogonal component. The first two components explained 17.3% and 12.9% of X variance, respectively, with R^2^X(cum) = 30.2%. Although the explained X variance was moderate, the model showed strong class‐explanatory and predictive performance, with R^2^Y(cum) = 87.1% and Q^2^(cum) = 77.2% (model‐selection results and additional validation results are provided in Supplementary Table S1, Figure S1 and Figure S2, respectively). In the score plot, the immature peel samples from Anhui, Guangdong, and Sichuan reported by T.‐X. Li et al. (2026) separated mainly along Component 1. In contrast, the leaf samples reported by Karioti et al. (2007) and Kasali et al. (2010) separated from peel‐related samples mainly along Component 2 (Figure 3). This multivariate separation was consistent with the tissue‐level composition plots and the heatmap patterns, in which peel and immature peel samples were Limonene‐dominant whereas leaf samples were enriched in sabinene, γ‐terpinene, β‐ocimene, linalool, and related monoterpenes (Figures 1A–C and 2).

**FIGURE 4:**
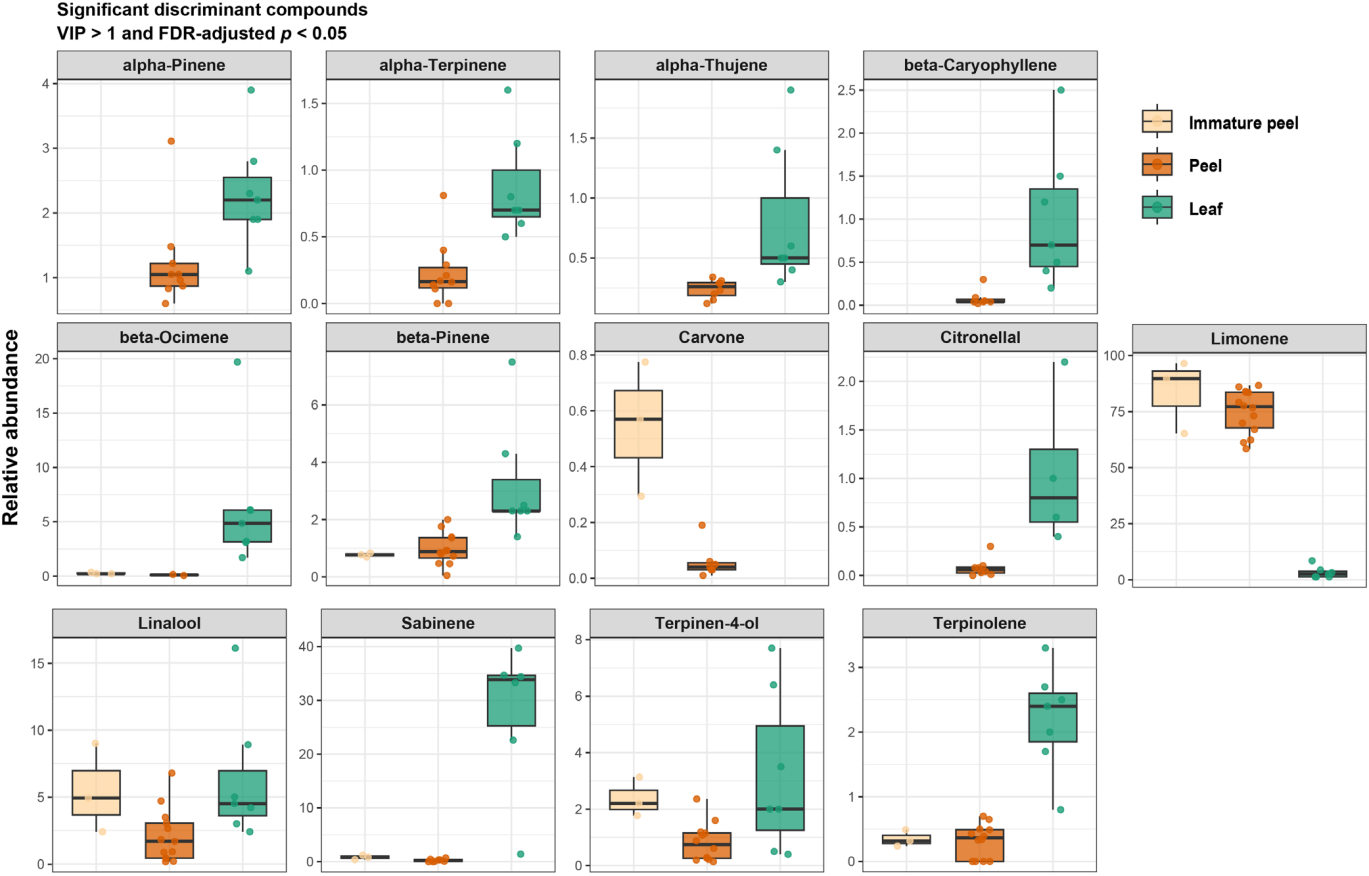
Distribution of significant discriminant compounds identified by integrated PLS‐DA and univariate analysis. Boxplots show the normalized relative abundance of 13 discriminant compounds that satisfied VIP > 1 and FDR‐adjusted *p* < 0.05 and showed large Kruskal‐Wallis *ε* ^2^ effect sizes, ranging from 0.290 to 0.772, indicating substantial tissue‐associated differences. Points represent individual samples. Limonene was enriched in peel and immature peel samples, whereas sabinene, terpinolene, α‐terpinene, β‐caryophyllene, β‐pinene, α‐thujene, α‐pinene, citronellal, β‐ocimene, linalool, and terpinen‐4‐ol were mainly enriched in leaf samples. Carvone was mainly associated with immature peel. These compounds collectively explain the tissue‐dependent separation observed in the PLS‐DA model. Because all compounds displayed in the figure already met the stated significance and effect‐size criteria, additional significance symbols were not included. Free y‐axis scales were used to clearly display compounds with markedly different relative‐abundance ranges.

The integrated biomarker analysis identified 13 significant discriminant compounds that satisfied VIP > 1 and FDR‐adjusted *p* < 0.05 and showed large Kruskal‐Wallis *ε* ^2^ effect sizes (Table 1). Limonene was the strongest peel‐related discriminant compound, with the highest VIP value (2.26) and a large KW *ε* ^2^ value (0.625). Sabinene showed the largest KW *ε* ^2^ value (0.772) and was strongly enriched in leaf samples, supporting its role as a major leaf‐associated marker. β‐Ocimene also showed a large tissue effect (KW *ε* ^2^ = 0.739) and was strongly leaf‐enriched, although its lower VIP value (1.08) suggests that it contributed less to the multivariate model than Limonene, terpinolene, or sabinene. Terpinolene, β‐pinene, α‐terpinene, α‐thujene, α‐pinene, β‐caryophyllene, citronellal, linalool, and terpinen‐4‐ol also supported the separation of leaf from peel‐related samples. Carvone was mainly enriched in immature peel and therefore represented the clearest immature peel‐associated discriminant compound (Figure 4; Table 1).

Cultivar identity also contributed to compositional variation. In the L. Li peel cultivar dataset, Limonene ranged from 58.38% in Qingpi to 83.49% in Hongmeiren. Qingpi showed high δ‐3‐carene (12.66%), whereas Nanfeng showed relatively high γ‐terpinene (8.48%) and linalool (6.78%) (L. Li, Jiang, et al. 2023). These patterns indicate that cultivar or genotype can strongly affect both major monoterpenes and lower‐abundance aroma‐active compounds.

Fruit maturity affected the peel volatile profile as well. In the Chachi maturity‐series samples, normalized Limonene increased from 67.57% in immature peel to 78.80% in semi‐mature peel and 80.60% in mature peel. Over the same sequence, γ‐terpinene decreased from 15.45% in immature peel to 11.34% in semi‐mature peel and 12.42% in mature peel, while p‐cymene declined from 5.80% to 2.67% and 1.53%, respectively (J. Cao et al. 2024). These patterns suggest developmental regulation of monoterpene accumulation and transformation during peel maturation.

Geographic variation was evident in the immature peel samples from Anhui, Guangdong, and Sichuan. In the original table, these samples were reported in mg/g, and Limonene concentrations were 96.468, 89.698, and 65.246 mg/g, respectively (T.‐X. Li et al. 2026). After normalization, Limonene accounted for 68.06%–74.42% of the immature peel composition. Other immature peel constituents also varied geographically, including linalool (2.74%–6.35%), o‐cymene (4.63%–7.27%), γ‐terpinolene (2.46%–3.10%), terpinen‐4‐ol (1.69%–2.21%), and myrcene (1.68%–1.85%) (T.‐X. Li et al. 2026). These differences suggest that geographic origin may influence the volatile profile of immature peel‐derived CrEO.

Overall, this analysis indicates that CrEO is a chemically flexible and strongly tissue‐dependent essential oil system. Peel‐derived and immature peel oils are generally limonene‐dominant, whereas leaf‐derived oils are more chemically diverse and often enriched in sabinene, γ‐terpinene, β‐ocimene, linalool, δ‐3‐carene, and p‐cymene. Therefore, CrEO should be described using tissue‐, cultivar‐, maturity‐, and geography‐specific chemotype profiles rather than as a single invariant chemical composition.

## 3 Extraction and Processing of CrEO

Given that the chemical profile of CrEO is strongly influenced by extraction conditions and raw material characteristics, understanding the extraction and processing methods is essential for controlling its composition and functional properties.

CrEO is obtained through various extraction techniques, which strongly influence both yield and chemical composition. Hydrodistillation and steam distillation are among the most commonly used methods for extracting *C. reticulata* essential oil (Rehman et al. 2007). These techniques consistently produce oils rich in monoterpene hydrocarbons (Rehman et al. 2007; H. Wang et al. 2012). Limonene is the main compound in peel‐derived oils (Cui et al. 2021; Y. C. Zhang et al. 2026). This compound (limonene) is consistently dominant across different cultivars and locations (Mahmood et al. 2017; Dias et al. 2020). The efficiency of these processes can be significantly improved through optimization of operational parameters, including liquid‐to‐solid ratio, soaking time, and extraction duration, as demonstrated using response surface methodology (Cui et al. 2021). Meanwhile, mechanical techniques, such as cold pressing or hand pressing, are also applied, offering advantages in preserving thermolabile volatile compounds and maintaining characteristic aroma profiles (Dharmawan et al. 2008; Faulhaber et al. 1997).

The quality and compositional variability of CrEO is strongly influenced by raw material characteristics and processing conditions. Variations in cultivar, plant part (peel, leaf, flower, or branch), and geographical origin can lead to significant differences in oil yield, physicochemical properties, and bioactive composition (Merle et al. 2004; Y. C. Zhang et al. 2026; Mahmood et al. 2017). For instance, peel oils generally contain higher concentrations of limonene (Rehman et al. 2007), whereas leaf oils may be richer in γ‐terpinene or linalool (Rehman et al. 2007; Y. C. Zhang et al. 2026), reflecting tissue‐specific biosynthesis. Additionally, environmental factors such as altitude, temperature, and regional growing conditions further modulate the relative abundance of key constituents. These observations indicate that both genetic and ecological factors collectively shape the chemical profile of CrEO (Mahmood et al. 2017).

Beyond extraction, post‐extraction processing techniques are critical for enhancing the stability and functional performance of CrEO. Techniques such as fractionation and selective concentration of terpene components can improve storage stability and enable tailoring of the oil for specific applications. Moreover, the stage of oil collection during distillation has been shown to influence both chemical composition and biological activity, with variations observed in properties such as insecticidal and repellent efficacy (Y. C. Zhang et al. 2026). These findings highlight the importance of controlling both extraction and downstream processing parameters to achieve oils with consistent quality and targeted functionality.

In summary, the volatile profile and flavor quality of citrus essential oils from orange and tangor were influenced mainly by the extraction method, although certain compounds ((E)‐ocimene, α‐terpinene, and α‐terpinolene) were affected by species (Park et al. 2024). Hydrodistillation produced distinct chemical changes and promoted off‐flavor compounds more markedly in orange than in tangor. Therefore, careful selection of the extraction method is essential to optimize essential oil yield and flavor quality for industrial applications (Cui et al. 2021; Y. C. Zhang et al. 2026; Mahmood et al. 2017).

## 4 Biological Activities

The chemical composition of CrEO, characterized by a diverse profile of terpene‐rich volatile compounds, is closely influenced by extraction and processing techniques, which determine both its yield and functional quality. Variations in methods such as hydrodistillation, cold pressing, and advanced extraction technologies can significantly affect the composition and stability of bioactive constituents. These compositional and processing‐dependent differences, in turn, play a critical role in shaping the biological activities of CrEO.

At the compound level, limonene, which is generally the dominant constituent, may contribute to anti‐inflammatory effects by reducing the production of pro‐inflammatory mediators (Chi et al. 2013). γ‐Terpinene may contribute to antioxidant activity through the inhibition of lipid peroxidation, whereas sabinene has demonstrated concentration‐dependent antioxidant and antimicrobial effects (Foti and Ingold 2003; S. Sharma et al. 2019). Linalool provides an example of an oxygenated monoterpene that may contribute to antimicrobial and antifungal activities, partly through disruption of microbial membrane integrity (Li, Wang, et al. 2023).

Nevertheless, the biological effects of CrEO cannot be attributed to a single major constituent, because minor compounds and synergistic or antagonistic interactions may influence the activity of the complete oil. Reported CrEO has many beneficial effects, including antioxidant, antimicrobial, antifungal, antidiabetic, anti‐inflammatory, antiaging, anticancer, and insecticidal properties, making it useful in food, medicine, cosmetics, and agriculture (Wolffenbüttel et al. 2015; Ishfaq et al. 2021; Xu et al. 2026). To provide an integrated overview, the major biological activities of CrEO, together with their reported evaluation models, proposed mechanisms, potential applications, and representative references, are summarized in Table 2.

**TABLE 2:** Reported biological activities, mechanisms, and potential applications of CrEO.

Biological activity	Key evidence and proposed mechanisms	Potential applications	Representative references
Antioxidant activity	CrEO showed radical scavenging, reducing, metal‐chelating, and lipid peroxidation‐inhibitory activities in DPPH, ABTS, FRAP, metal‐chelating, and lipid oxidation assays. These effects are mainly associated with terpene constituents, particularly limonene and related oxygenated derivatives.	Food preservation, pharmaceutical formulations, and functional products	Shaw et al. (2023), Ariani et al. (2022), Mehmood et al. (2013), C. L. Li, Cai, et al. (2022), Sadaf et al. (2009), Boudries et al. (2017), Kunová et al. (2019)
Antimicrobial activity	CrEO inhibited both Gram‐positive and Gram‐negative bacteria, including *Staphylococcus* spp., *Escherichia coli* , *Bacillus subtilis* , *Listeria monocytogenes* , MRSA, *Listeria innocua* , and *Corynebacterium* species, mainly through membrane disruption, increased permeability, metabolic interference, and biofilm inhibition.	Natural antimicrobial agents for food preservation and pharmaceutical formulations	Ariani et al. (2022), Kacániová et al. (2019), W. L. Zhang et al. (2022); Peng et al. (2024), Boudries et al. (2017), Kunová et al. (2019)
Antifungal activity	CrEO showed inhibitory effects against *Candida albicans* and *Penicillium* species, possibly by inducing hyphal damage, leakage of intracellular components, and alteration of membrane lipid composition.	Control of fungal contamination in food and pharmaceutical products	Boudries et al. (2017), Tao et al. (2014)
Antidiabetic activity	CrEO exhibited inhibitory effects on α‐glucosidase and may contribute to postprandial glycaemic control through antioxidant and enzyme‐modulating mechanisms.	Functional ingredients or complementary agents for metabolic regulation	Mehmood et al. (2013), Aumeeruddy‐Elalfi et al. (2018)
Anti‐inflammatory activity	CrEO suppressed pro‐inflammatory mediators, including IL‐6, COX‐2, and NF‐κB, thereby mitigating inflammatory responses in experimental systems.	Prevention or management of inflammation‐related conditions	Li, Cai, et al. (2022)
Anti‐aging and cosmeceutical activities	CrEO inhibited collagenase, elastase, and acetylcholinesterase and reduced melanin production in melanocyte models, supporting anti‐aging and antimelanogenic potential.	Skin anti‐aging, depigmentation, and cosmetic formulations	Aumeeruddy‐Elalfi et al. (2018), Fahmy et al. (2022)
Anticancer and cytotoxic activities	CrEO induced G0/G1 cell‐cycle arrest and exerted cytotoxic effects in different cell lines, which may be related to terpene constituents such as limonene.	Cancer prevention‐related research and potential adjunct applications	Li, Cai, et al. (2022), Shaw et al. (2023), Wolffenbüttel et al. (2015)
Insecticidal and larvicidal activities	CrEO showed activity against *Callosobruchus maculatus* , *Sitophilus zeamais* , and mosquito larvae such as *Culex pipiens* , possibly through acetylcholinesterase and glutathione‐transferase inhibition and disruption of insect physiological functions.	Eco‐friendly pesticides, stored‐grain protection, mosquito control, and by‐product valorization	Dutra et al. (2016), Fouad and da Camara (2017), Oliveira et al. (2023), Shaw et al. (2023), Badawy et al. (2018), Xu et al. (2026)

### 4.1 Antioxidant Activity

Among the various bioactivities, antioxidant activity is one of the most consistently reported functions of CrEO. It exhibits strong radical scavenging capacity, as demonstrated by DPPH and related assays (Shaw et al. 2023) (Ariani et al. 2022). Significant antioxidant effects have also been confirmed through ABTS and FRAP assays, along with metal‐chelating activity (Mehmood et al. 2013). In addition, CrEO shows inhibition of lipid peroxidation (C. L. Li, Cai, et al. 2022) as reflected in a 49.98% inhibition of linoleic acid oxidation (Sadaf et al. 2009). These antioxidant effects are primarily attributed to its terpene constituents, particularly limonene and related oxygenated derivatives, which play a critical role in mitigating oxidative stress and maintaining cellular redox homeostasis (Shaw et al. 2023; C. L. Li, Cai, et al. 2022).

Notably, peel‐derived CrEO has been shown to exhibit significant antioxidant activity, particularly through DPPH radical scavenging and reducing power assays, with effects observed in a dose‐dependent manner (Boudries et al. 2017). Comparative analyzes indicate that mandarin ( *C. reticulata* ) essential oil displays stronger antioxidant capacity than other citrus varieties such as clementine and wilking, likely due to its high limonene content (77%–97%) and associated bioactive compounds (Boudries et al. 2017). In addition, studies involving multiple plant essential oils have confirmed that peel‐derived CrEO possesses measurable antioxidant potential, although its activity may vary relative to other species depending on compositional differences (Kunová et al. 2019).

Overall, these findings reinforce the role of CrEO as an effective natural antioxidant and support its potential application in food preservation, pharmaceutical formulations, and functional products aimed at reducing oxidative damage.

### 4.2 Antimicrobial and Antifungal Activities

In addition to its antioxidant potential, CrEO exhibits significant antimicrobial and antifungal activities against a broad spectrum of microorganisms. It has been shown to inhibit both Gram‐positive and Gram‐negative bacteria, including *Staphylococcus* spp., *Escherichia coli* , *Bacillus subtilis* , and *Listeria monocytogenes* , primarily through mechanisms involving disruption of cell membrane integrity, increased permeability, and interference with biofilm formation (Ariani et al. 2022; Kacániová et al. 2019; W. L. Zhang et al. 2022; Peng et al. 2024).

Notably, peel derived CrEO has demonstrated pronounced antibacterial activity against a range of pathogenic and spoilage microorganisms. It exhibits strong inhibitory effects against *Escherichia coli* , *Staphylococcus aureus* , methicillin‐resistant *S. aureus* (MRSA), *Listeria innocua* , and *Candida albicans* , with measurable inhibition zones and low minimum inhibitory concentrations, indicating high efficacy (Boudries et al. 2017). Similarly, CrEO has shown inhibitory activity against multiple Gram‐positive and Gram‐negative bacterial strains, including Corynebacterium species, further confirming its broad‐spectrum antimicrobial potential (Kunová et al. 2019). These activities are largely attributed to its high limonene content and other bioactive volatile compounds, which contribute to membrane disruption and metabolic interference in microbial cells.

Furthermore, antifungal activity against Penicillium species has also been reported, where CrEO induces structural damage to fungal hyphae, promotes leakage of intracellular components, and alters membrane lipid composition, ultimately leading to cell death (Tao et al. 2014). These findings support the applicability of CrEO as a natural antimicrobial agent in food preservation and pharmaceutical formulations aimed at controlling microbial contamination and extending product shelf life.

### 4.3 Antidiabetic and Anti‐Inflammatory Activities

CrEO is increasingly recognized as a multifunctional bioactive agent with significant potential in pharmaceutical and cosmeceutical applications, owing to its metabolic regulatory, anti‐inflammatory, and antiaging properties. Growing evidence indicates that CrEO exhibits antidiabetic activity, which is likely associated with its antioxidant capacity and its ability to modulate key enzymes involved in glucose metabolism (Mehmood et al. 2013). In particular, CrEO has demonstrated potent inhibitory effects against α‐glucosidase, a crucial enzyme in carbohydrate digestion, in some cases surpassing the efficacy of conventional antidiabetic drugs such as acarbose (Aumeeruddy‐Elalfi et al. 2018). These findings suggest that CrEO may contribute to the regulation of postprandial hyperglycaemia and metabolic homeostasis.

Beyond its metabolic effects, CrEO also exhibits significant anti‐inflammatory activity. This is primarily mediated through the suppression of pro‐inflammatory mediators, including interleukin‐6 (IL‐6), cyclooxygenase‐2 (COX‐2), and nuclear factor kappa B (NF‐κB), thereby mitigating inflammatory responses in experimental systems (C. L. Li, Cai, et al. 2022). Such mechanisms highlight its potential role in the prevention and management of chronic inflammatory conditions.

In addition, CrEO demonstrates notable antiaging effects, largely attributed to its inhibitory action on enzymes involved in skin matrix degradation. Specifically, CrEO has been shown to inhibit collagenase and elastase, which are responsible for the breakdown of collagen and elastin, key structural components of the skin. Furthermore, inhibition of acetylcholinesterase suggests additional relevance in delaying neurodegenerative processes (Aumeeruddy‐Elalfi et al. 2018). Enzyme kinetic studies indicate that these inhibitory effects may occur via both competitive and uncompetitive mechanisms, reflecting the complex interactions between CrEO constituents and target enzymes.

Moreover, CrEO exhibits antimelanogenic activity by effectively reducing both intracellular and extracellular melanin production in melanocyte models, further supporting its application in skin depigmentation and cosmetic formulations (Aumeeruddy‐Elalfi et al. 2018). Collectively, these diverse bioactivities underscore the therapeutic versatility of CrEO and support its development as a natural, sustainable alternative to synthetic agents for the management of metabolic disorders, inflammation, and skin aging, while enhancing its value in pharmaceutical and cosmetic industries (Fahmy et al. 2022).

### 4.4 Anticancer and Cytotoxic Activities

Recent studies have increasingly focused on the anticancer and cytotoxic properties of CrEO. It has been reported to induce cell cycle arrest at the G0/G1 phase and exert cytotoxic effects on various cell lines, suggesting potential applications in cancer prevention and adjunct therapy (C. L. Li, Cai, et al. 2022). These bioactivities are closely linked to its terpene constituents, particularly limonene, which has been widely reported for its chemopreventive and pharmacological effects (Shaw et al. 2023; Wolffenbüttel et al. 2015).

## 5 Volatile Profile of *C. reticulata*

CrEO is derived from the intrinsic volatile constituents of *C. reticulata* , which are primarily localized in peel oil glands, where diverse alkenes, alcohols, aldehydes, ketones, phenols, and terpenoid constituents accumulate (Yang et al. 2022). Therefore, the composition and quality of CrEO are closely governed by the plant's volatile profile, which is shaped by biochemical, developmental, genetic, and environmental factors. Changes in volatile concentration patterns during fruit development have also been linked to metabolic regulation and cultivar‐dependent differences (J. Wang et al. 2022).

*C. reticulata* exhibits a chemically diverse profile comprising both non‐volatile phytochemicals and volatile compounds that jointly determine their functional and sensory characteristics. The non‐volatile constituents include major flavonoids such as hesperidin and polymethoxyflavones (e.g., nobiletin and tangeretin), as well as phenolic acids, synephrine, and limonin, whose levels can decline or transform during storage (Chao et al. 2022; Ferreira et al. 2018). Other types of compounds, including coumarins, lignins, alkaloids, and amino acids, have also been found, showing that *C. reticulata* has a complex chemical composition that changes with factors like tree age and genetics (K. X. Cao et al. 2026; Y. Y. Wang, Huang, et al. 2025).

The volatile fraction is dominated by terpene hydrocarbons, particularly limonene, which is consistently reported as the principal compound in both peel and juice (Barboni et al. 2011). Other important terpenes such as γ‐terpinene, β‐myrcene, and α‐pinene contribute significantly to the characteristic citrus aroma of mandarin peels (Elmaci and Onogur 2012). In the pericarp essential oil of “Chachi,” monoterpene hydrocarbons predominate, with limonene as the major component and oxygenated minor constituents such as linalool, citral, terpinen‐4‐ol, trace esters, and phenolic derivatives contributing to its citrus‐like and mildly herbaceous aroma profile (Chao et al. 2022).

Oxygen‐containing aroma‐active compounds, including aldehydes, alcohols, esters, and ketones, further contribute to aroma differentiation across maturity stages and product types (Wang, Wang, et al. 2023). Gas chromatography‐olfactometry studies have shown that aldehydes and monoterpenes, together with esters, alcohols, and ketones, constitute major aroma‐active groups, with compounds such as 1,8‐cineole, beta‐myrcene, (E,E)‐2,4‐nonadienal, hexanal, ethyl 2‐methylbutanoate, and linalool strongly influencing sensory perception (Miyazaki et al. 2012). Importantly, the characteristic tangerine flavor is not determined by a single compound but by specific combinations of volatile constituents that generate distinct sensory attributes across hybrids and cultivars (Miyazaki et al. 2012).

Minor and trace aroma‐active compounds also contribute to aroma complexity. Methyl anthranilate derivatives and alpha‐phellandrene have been identified as important contributors to flavor perception and quality during aging (Liu et al. 2024), while unsaturated aldehydes further enrich the overall aroma profile (Naef and Velluz 2001). Comprehensive profiling studies have confirmed that *C. reticulata* volatiles include terpenes, carbonyls, alcohols, esters, and hydrocarbons, with limonene remaining the dominant constituent across cultivars (Dharmawan et al. 2007).

The volatile composition of *C. reticulata* is highly dynamic and is influenced by fruit development, ripening, processing, storage, and genetic background. Drying and other processing methods can alter volatile retention and favor the preservation or loss of specific compound classes, with terpenes and esters often serving as key differentiating metabolites (X. Y. Yu et al. 2022; M. Wang, Li, et al. 2023). Similar variation is evident in juice, where volatile levels depend on extraction method and maturity stage (Barboni et al. 2010). Together, these factors shape the aroma quality, functional potential, and application value of CrEO (Y. Yu et al. 2018).

## 6 Applications for *C. reticulata* Peels and Peel‐Derived Essential Oils

The byproducts of *C*., particularly peels and their derived essential oils, have attracted considerable attention due to their high content of bioactive compounds and their potential for sustainable valorization. Current applications span the food, beverage, cosmetic, pharmaceutical, oral healthcare, and agricultural sectors. The major application categories are discussed below.

### 6.1 Food and Beverage Applications

Citrus peels are rich in polyphenols, flavonoids, dietary fibers, and essential oils, which confer strong antioxidant and antimicrobial properties (Velásquez‐Rivera and Díaz‐Torres 2024). These characteristics support their application as functional ingredients in food systems, especially in meat products, where they can enhance oxidative stability, extend shelf life, and improve sensory and physicochemical properties (Velásquez‐Rivera and Díaz‐Torres 2024). Furthermore, peel‐derived products such as citrus‐based liquors demonstrate high aromatic complexity and antioxidant capacity, highlighting their potential in the development of value‐added food and beverage products while contributing to waste reduction and economic sustainability (Navarro‐Martínez et al. 2019).

### 6.2 Cosmeceutical Applications

Peel derived CrEO exhibits diverse biological activities associated with their terpene‐rich composition. These oils possess significant antioxidant, anti‐aging, and anti‐melanogenic properties, primarily through the inhibition of enzymes such as collagenase and tyrosinase, making them promising candidates for cosmeceutical applications (Prommaban and Chaiyana 2022). Advances in formulation strategies, including microemulsion systems, have further improved their stability and reduced irritation, enhancing their applicability in products for external use (Prommaban and Chaiyana 2022).

### 6.3 Pharmaceutical and Oral Healthcare Applications

Preliminary evidence also indicates that CrEO may have applications beyond the cosmetic sector. Its reported effects on phosphodiesterase type 5 (PDE5) and Rho‐associated protein kinase II (ROCK‐II) suggest possible applications in managing erectile dysfunction (Hamdy et al. 2025). In addition, nano‐encapsulation approaches have significantly enhanced their antibacterial, antibiofilm, and antioxidant activities, particularly against oral pathogens like *Streptococcus mutans* , supporting their incorporation into oral healthcare formulations (Torshabi et al. 2023).

### 6.4 Insecticidal and Larvicidal Application

CrEO exhibits promising potential in agricultural and environmental applications due to its insecticidal and larvicidal activities. Notably, CrEO has demonstrated efficacy in controlling the cowpea weevil ( *Callosobruchus maculatus* ), a major pest of stored cowpea grains that significantly compromises post‐harvest quality and food security, thereby underscoring the urgent need for safer and more sustainable alternatives to conventional chemical pesticides (Dutra et al. 2016). Moreover, the insecticidal activity of CrEO and both enantiomers of their constituent, limonene [(R)‐(+)‐ and (S)‐(−)‐limonene], was assessed against *Sitophilus zeamais* under laboratory conditions (Fouad and da Camara 2017).

In addition to its activity against storage pests, CrEO exhibits potent larvicidal effects against mosquito species such as *Culex pipiens* , with mechanisms involving inhibition of key enzymes (e.g., acetylcholine esterase and glutathione‐transferase) and disruption of normal physiological functions in insects (Oliveira et al. 2023; Shaw et al. 2023; Badawy et al. 2018). These properties position CrEO as a viable eco‐friendly alternative to synthetic pesticides. However, its efficacy, safety, environmental persistence, and effects on non‐target organisms require validation under field conditions.

### 6.5 Emerging Anti‐Fatigue, Digestive, and Emotion‐Regulating Effects

Emerging evidence indicates that CrEO may influence cellular pathways related to physical fatigue. Essential oil obtained from the peel waste of *C. reticulata* var. *depressa* increased ATP content, mitochondrial membrane potential, and mitochondrial mass in C2C12 skeletal muscle cells. It also activated the AMPK/SIRT1/PGC‐1α pathway and increased the expression of mitochondrial biogenesis‐related factors. γ‐Terpinene was identified as a major contributor to these effects (C. H. Wang, Yeo, et al. 2025). These findings provide a possible mechanistic basis for anti‐fatigue activity; however, because the evidence was obtained using a cellular model, confirmation through animal and human studies is still required.

Volatile constituents derived from *C. reticulata* peel may also contribute to digestive‐function improvement. Volatile fractions from different aged Citri Reticulatae Pericarpium promoted gastric emptying and small‐intestinal propulsion in a spleen‐deficiency rat model, with the observed activity attributed to the combined effects of multiple constituents (Lv Lv et al. 2024). In addition, volatile oil obtained from immature *C. reticulata* peel increased fecal output, fecal water content, and small‐intestinal propulsion in mice with loperamide‐induced slow‐transit constipation. The proposed mechanisms involved regulation of gastrointestinal hormones, activation of SCF/c‐Kit signaling, and modulation of the intestinal microbiota (Zou et al. 2025). Although these findings support potential gastrointestinal benefits, they remain preclinical and should not be interpreted as evidence of clinical efficacy.

CrEO has additionally shown preliminary emotion‐regulating effects in animal models. Peel essential oil from *C. reticulata* var. *depressa* improved anxiety‐ and depression‐like behaviors in chronically stressed mice, potentially through modulation of the gut–brain axis and intestinal microbiota (Tsai et al. 2023). A separate study found that intranasally administered *C. reticulata* essential oil produced a rapid antidepressant‐like effect in mice. A mixture containing linalool, *p*‐cymene, α‐terpinene, terpinen‐4‐ol, and α‐terpineol exhibited both antidepressant‐ and anxiolytic‐like effects, suggesting that oxygenated and hydrocarbon terpenes may act through multiple neuroactive and inflammatory pathways (Khanh Nguyen et al. 2024). Nevertheless, these findings are based primarily on animal, cellular, and computational models, and clinical studies are needed to determine their relevance to human emotional health.

### 6.6 Sustainable Valorization and Future Application Perspectives

The utilization of citrus peel waste as a raw material for essential oil extraction aligns with principles of sustainability and circular bioeconomy, contributing to the valorization of agro‐industrial by‐products while reducing environmental burden (Xu et al. 2026). Overall, the valorization of *C. reticulata* peels and essential oils represents a promising strategy for transforming agro‐industrial waste into high‐value products with applications across the food, pharmaceutical, and cosmetic industries, while contributing to sustainability and circular economy approaches.

## 7 Conclusion and Future Perspectives

CrEO should not be regarded as an independent chemical entity, but as a concentrated representation of the peel‐derived constituents of *C. reticulata* , particularly those biosynthesized, stored, and released from peel oil glands. Although *C. reticulata* contains both non‐volatile and volatile phytochemicals, the composition and quality of CrEO are governed primarily by the fruit's volatile profile. As a chemically diverse species, *C. reticulata* contains metabolites that collectively shape its functional attributes and sensory character. Accordingly, the characteristic aroma of *C. reticulata* results from complex interactions among multiple volatile constituents rather than a single defining compound (Miyazaki et al. 2012).

Beyond its nutritional value, *C. reticulata* offers significant potential for byproduct valorization, particularly through its peels, which are rich in polyphenols, essential oils, and dietary fibers with strong antioxidant and antimicrobial activities. These properties support their application in functional foods, such as meat products, where they enhance shelf life and quality while promoting sustainability (Velásquez‐Rivera and Díaz‐Torres 2024). Peel‐derived products, including citrus‐based liquors, further demonstrate high aromatic and commercial potential (Navarro‐Martínez et al. 2019).

Essential oils from *C. reticulata* exhibit diverse bioactivities, including antioxidant, antimicrobial, anti‐aging, and enzyme‐inhibitory effects, supporting their use in cosmeceutical, pharmaceutical, and oral care applications (Prommaban and Chaiyana 2022; Torshabi et al. 2023). Emerging therapeutic potentials have also been reported (Hamdy et al. 2025).

Future research should prioritize optimized extraction methods, understanding synergistic compound interactions, and scalable valorization strategies to fully support the integration of *C. reticulata* into sustainable, high‐value industrial applications.

## Author Contributions

**Jiaojiao Zhang:** conceptualization, visualization, funding acquisition, data curation, writing – original draft, writing – review and editing, investigation, methodology. **Peng Zhou:** investigation, writing – review and editing. **Yongxian Chen:** methodology. **Andrea Brenciani:** resources, writing – review and editing. **Jing Zhou:** resources, conceptualization, writing – review and editing. **Iman Zarei:** conceptualization, resources, writing – review and editing. **Amnah Nasim:** conceptualization, funding acquisition.

## Funding

This work was supported by the scientific research foundation of Zhejiang A&F University (2023CFR008), the international exchange program from the China Scholarship Council (CSC) (Grant No. 202408330353, Grant No. 202404910150), the Institute of Information & Communications Technology Planning & Evaluation (IITP)‐Innovative Human Resource Development for Local Intellectualization program grant funded by the Korea government (MSIT) (IITP‐2026‐RS‐2024‐00439292).

## Conflicts of Interest

The authors declare no conflicts of interest.

## Supporting information


**Figure S1:** PLS‐DA model validation. The PLS‐DA model validation figure (Figure S1) showed clear separation among the study groups in the first two latent components. The model displayed strong explanatory and predictive performance, with high cumulative R^2^Y and *Q* ^2^ values. Permutation testing supported the validity of the model, with significant *p*‐values for both R^2^Y and *Q* ^2^, indicating that the observed discrimination was unlikely to arise from random class assignment. Observation diagnostics showed that most samples were within acceptable limits, although a small number of samples may represent potential outliers or influential observations.
**Figure S2:** Compounds with VIP scores > 1 identified by PLS‐DA. Compounds with VIP scores greater than 1 were considered important contributors to group discrimination in the PLS‐DA model. Higher VIP scores indicate stronger contributions of individual volatile constituents to compositional variation among CrEO samples.
**Table S1:** shows that model performance was evaluated across one‐, two‐, and three‐component PLS‐DA models. The increase in Q2 from the one‐component to the two‐component model was substantial (ΔQ2 = 0.566), whereas the increase from the two‐component to the three‐component model was modest (ΔQ2 = 0.061). Thus, although the three‐component model slightly improved predictive performance, this gain was limited relative to the added model complexity. The two‐component model was therefore preferred, as it provided strong class discrimination and predictive performance while maintaining model parsimony.

## Data Availability

The data supporting the findings and secondary analyses presented in this review are available within the article and its Supporting Informations. All underlying data were derived from the published sources cited in the manuscript.
