# Supplementary material for: Citrus reticulata Essential Oil as a Bioactive Food Ingredient: Composition Variability, Extraction, Functional Bioactivities, and Cross‐Study Quantitative Analysis
Source: Food Sci Nutr. 2026 Sep 21;14(9):e72380. doi: 10.1002/fsn3.72380 (PMC13594552; doi:10.1002/fsn3.72380)
Supplement: Supplementary file 1 [file FSN3-14-e72380-s001.docx]

**Title: *Citrus reticulata* Essential Oil as a Bioactive Food Ingredient: Composition Variability, Extraction, Functional Bioactivities, and Cross-Study Quantitative Analysis**

Jiaojiao Zhang ^1, 2, #, *^, Peng Zhou ^7, #^, Yongxian Chen ^8^, Andrea Brenciani ^3, *^, Jing Zhou ^5, *^, Iman Zarei ^6^, Amnah Nasim ^4^

^1^ College of Food and Health, Zhejiang A& F University, Hangzhou 311300, China.

^2^ Biological Physics Group, University of Manchester, Schuster Building, Oxford Road, Manchester M13 9PL, United Kingdom.

^3^ Department of Biomedical Sciences and Public Health, Polytechnic University of Marche, 60126 Ancona, Italy

^4^ Jeonbuk RICE Intelligence Innovation Research Center, Jeonbuk National University, Jeonju, 54896, Jeonbuk, Republic of Korea

^5^ Agriculture and Rural Affairs Bureau of Qujiang District, Quzhou, Zhejiang, China

^6^ Institute of Public Health and Clinical Nutrition, School of Medicine, Faculty of Health Science, University of Eastern Finland, P.O. Box 1627, 70211, Kuopio, Finland.

^7^ Institute of Process Engineering, Chinese Academy of Sciences, Beijing 100190, P. R. China.

^8^ Tea Resources Utilization and Quality Testing Key Laboratory of Sichuan Province, Sichuan Agricultural University, Chengdu, 611130, China

*Correspondence: jjzhang@zafu.edu.cn (J.Z.); 15268059651@163.com (J.Z.); a.brenciani@univpm.it (A. B.)

**Supplementary Table 1.** Performance metrics and component selection for PLS-DA models

| Predictive components | Orthogonal components | R²X(cum) | R²Y(cum) | Q²(cum) | RMSEE | pR²Y | pQ² | ΔQ² vs previous model | Decision / interpretation |
| --- | --- | --- | --- | --- | --- | --- | --- | --- | --- |
| 1 | 0 | 0.173 | 0.388 | 0.206 | 0.378 | 0.04 | 0.01 | — | Not selected: limited explanatory power for tissue-group classification and low cross-validated predictive ability. |
| 2 | 0 | 0.302 | 0.871 | 0.772 | 0.17 | 0.01 | 0.01 | 0.566 | Selected: strong goodness-of-fit and predictive ability, with substantially lower estimation error and acceptable parsimony. |
| 3 | 0 | 0.4 | 0.931 | 0.833 | 0.12 | 0.01 | 0.01 | 0.061 | Not selected: metrics improved slightly, but the incremental gain was modest relative to the added model complexity and potential overfitting risk. |

**Table note.** R²X(cum), cumulative proportion of predictor-matrix variation explained by the model; R²Y(cum), cumulative proportion of tissue-group response variation explained by the model; Q²(cum), cross-validated predictive ability; RMSEE, root mean squared error of estimation; pR²Y and pQ², permutation-test p values for R²Y and Q², respectively. Candidate models containing one to three predictive components were initially screened using seven-fold cross-validation and 100 permutation tests. After model selection, the final two-component model was subjected to a more extensive validation using 200 permutation tests. Therefore, the permutation p-values reported for the selected model in Figure S1 differ from those presented in Table S1.

Supplementary Table 1 shows that model performance was evaluated across one-, two-, and three-component PLS-DA models. The increase in Q² from the one-component to the two-component model was substantial (ΔQ² = 0.566), whereas the increase from the two-component to the three-component model was modest (ΔQ² = 0.061). Thus, although the three-component model slightly improved predictive performance, this gain was limited relative to the added model complexity. The two-component model was therefore preferred, as it provided strong class discrimination and predictive performance while maintaining model parsimony.

**Supplementary Figure 1.** PLS-DA model validation

The PLS-DA model validation figure (Supplementary Figure 1) showed clear separation among the study groups in the first two latent components. The model displayed strong explanatory and predictive performance, with high cumulative R²Y and Q² values. Permutation testing supported the validity of the model, with significant *p*-values for both R²Y and Q², indicating that the observed discrimination was unlikely to arise from random class assignment. Observation diagnostics showed that most samples were within acceptable limits, although a small number of samples may represent potential outliers or influential observations.

**Supplementary Figure 2.** Compounds with VIP scores > 1 identified by PLS-DA

Compounds with VIP scores greater than 1 were considered important contributors to group discrimination in the PLS-DA model. Higher VIP scores indicate stronger contributions of individual volatile constituents to compositional variation among CrEO samples.
